# Survival advantage of partial over radical nephrectomy in patients presenting with localized renal cell carcinoma

**DOI:** 10.1186/1471-2407-14-372

**Published:** 2014-05-26

**Authors:** Frederik C Roos, Sandra Steffens, Kerstin Junker, Martin Janssen, Frank Becker, Gerd Wegener, Walburgis Brenner, Julie Steinestel, Thomas J Schnoeller, Mark Schrader, Rainer Hofmann, Joachim W Thüroff, Markus A Kuczyk, Heiko Wunderlich, Stefan Siemer, Arndt Hartmann, Michael Stöckle, Andres J Schrader

**Affiliations:** 1 Department of Urology, Mainz University Medical Center, Mainz, Germany; 2 Department of Urology and Urological Oncology, Medical School Hannover, Hannover D-30625, Germany; 3 Department of Urology, Saarland University Medical Center, Homburg, Saar, Germany; 4 Department of Urology, Jena University Hospital, Jena, Germany; 5 Boxberg Centre, Urological Group and Clinic Derout/Pönicke/Becker, Neunkirchen, Germany; 6 Cancer Center, Hannover University Medical School, Hannover, Germany; 7 Department of Urology, Ulm University Medical Center, Ulm, Germany; 8 Department of Urology, Philipps University of Marburg, Marburg, Germany; 9 Clinic of Urology and Pediatric Urology, Eisenach St. Georg Hospital, Eisenach, Germany; 10 Department of Pathology, Erlangen University Medical Center, Erlangen, Germany

## Abstract

**Background:**

Partial nephrectomy (PN) preserves renal function and has become the standard approach for T1a renal cell carcinoma (RCC). However, there is still an ongoing debate as to which patients will actually derive greater benefit from partial than from radical nephrectomy (RN). The aim of this study was to retrospectively evaluate the impact of the type of surgery on overall survival (OS) in patients with localized RCC.

**Methods:**

Renal surgery was performed in 4326 patients with localized RCC (pT ≤ 3a N/M0) at six German tertiary care centers from 1980 to 2010: RN in 2955 cases (68.3%), elective (ePN) in 1108 (25.6%), and imperative partial nephrectomy (iPN) in 263 (6.1%) cases. The median follow-up for all patients was 63 months. Kaplan-Meier and Cox regression analyses were carried out to identify prognosticators for OS.

**Results:**

PN was performed significantly more often than RN in patients presenting with lower tumor stages, higher RCC differentiation, and non-clear cell histology. Accordingly, the calculated 5 (10)-year OS rates were 90.0 (74.6)% for ePN, 83.9 (57.5)% for iPN, and 81.2 (64.7)% for RN (p < 0.001). However, multivariate analysis including age, sex, tumor diameter and differentiation, histological subtype, and the year of surgery showed that ePN compared to RN still qualified as an independent factor for improved OS (HR 0.79, 95% CI 0.66-0.94, p = 0.008).

**Conclusion:**

Even allowing for the weaknesses of this retrospective analysis, our multicenter study indicates that in patients with localized RCC, PN appears to be associated with better OS than RN irrespective of age or tumor size.

## Background

Complete surgical excision of the tumor still remains the only curative treatment for renal cell carcinoma (RCC) [1]. Preserving renal function by performing partial nephrectomy (PN), was originally reserved for patients with an anatomically or functionally solitary kidney or for those with a functioning contralateral kidney at risk for future functional impairment [2]. However, the use of PN has increased tremendously, even in patients with localized unilateral RCC and a healthy contralateral kidney [3]. Having shown excellent long-term oncological outcomes equivalent to those of radical nephrectomy (RN) [4-7], combined with limited perioperative morbidity [8], PN has become the gold standard for all patients with renal tumors < 4 cm [1,6,9,10]. Some authors recommend PN in all cases where PN is oncologically safe and technically feasible, even for pT ≥ 1b and high-risk tumors [10-13]. This is surely attributable in part to recent studies demonstrating that elective PN (ePN) can be associated with significantly lower long-term mortality than RN [14-17], probably due to the preservation of renal function [18-20] and the lower incidence of subsequent cardiovascular diseases (CVD) [14].

While it is indisputable that PN leads to better preservation of renal function, there is still debate over the extent to which this surgically induced chronic kidney disease does also increase the risk of CVD and non-RCC-related death [21-23]. This observation became a particularly hot issue after van Poppel et al. [24] published the overall survival (OS) results of the EORTC 30904 phase III study. Contrary to expectations, the authors found no OS advantage of ePN over RN.

In view of these contradictory results, this large retrospective multicenter study was performed to comparatively investigate partially and radically nephrectomized patients comprising tumor and patient parameters and to evaluate the influence of the surgical technique on OS of patients with localized RCC.

## Methods

### Patient selection and tumor characteristics

This study included 4326 patients who underwent surgery for localized RCC (pT1-3a, no detectable metastasis at the time of surgery) between 1980 and 2010 at Homburg (n = 1200), Mainz (n = 911), Hannover (n = 647; 1991–2005), Ulm (n = 495; 1998–2010), Jena (n = 597) or Marburg (n = 476; 1990–2005) University Medical Centers. Preoperative staging included CT scan in most cases. Selection of patients for PN was based on tumor size and location as well as on discussions and approval by tumor boards at each center and/or the patient’s or surgeon’s preference. PN was defined as “imperative” in case of significant preexisting renal insufficiency (GFR < 60 ml/min) and/or the absence of a normal contralateral kidney. However, eventually the definition of an “imperative” indication was based in every individual case on the personal judgment of the operating surgeon.

Staging was based on the 2002 TNM classification system. Institutional databases provided information on patient and tumor characteristics. The primary end point of this study was OS. The ethics committees of each institution (Ethics Committee of the Medical School Hannover; Ulm University Medical Center; State Chamber of Physicians Rheinland-Pfalz, Germany; Jena University Hospital and State Chamber of Physicians Saarland) approved the study.

### Statistical methods

Continuous variables were reported as mean values and standard deviations (SD) for parametric distributions or as median values and interquartile ranges (IQR) for non-parametric distributions. Chi-square or Fisher’s exact tests were conducted to assess differences in covariate distributions between patients treated by PN and those who underwent RN. Kaplan-Meier estimates of survival time were calculated, and subgroups were compared by the log rank test. Multivariate Cox regression models were used to assess the association between survival and the chosen surgical procedure adjusted for different patient and tumor covariates. SPSS 19.0 was used for statistical assessment. In all tests, a two-sided p < 0.05 was considered to indicate significance.

## Results

Our patient population of 2675 (61.8%) men and 1651 (38.2%) women had a mean (median) age of 61.2 (62.0) years (range, 16–92). 3545 (81.9%) had clear cell, 496 (11.5%) papillary, 182 (4.2%) chromophobe, and 103 (2.3%) unclassified RCC. There were 3259 (75.3%) patients with pT1, 530 (12.3%) with pT2, and 537 (12.4%) with pT3a RCC. The mean (median) tumor size was 4.9 (4.3) cm (SD ±2.7 cm).

The tumor grade was G1 in 1020 (24.5%), G2 in 2705 (65.0%), G3 in 420 (10.1%), and G4 in 16 (0.4%) of all evaluable patients (Table 1). The mean (median) follow-up for all patients was 74 (63) months (IQR: 30–109 months). It did not differ significantly between patients treated with RN (mean, 75 months) and elective PN (ePN; mean, 72 months, p = 0.07, t-test), but was shorter in those who received an imperative PN (iPN; mean, 65 months, p = 0.003, t-test). By the last day of data acquisition, 1061 (24.5%) had died of RCC or other causes.

**Table 1:** Association between different patient and tumor parameters according to surgical procedure

**Variable**	**RN**	**ePN**	**iPN**	**p-value**	**Test**
Age ± SD, mean [years]^1^	61.6 ± 11.1	59.7 ± 11.6	62.8 ± 11.2	< 0.001	ANOVA
Sex				< 0.001	chi-square
Female	1186 (40.1%)	360 (32.5%)	105 (39.9%)		
Male	1769 (59.9%)	748 (67.5%)	158 (60.1%)		
Tumor diameter ± SD, mean [cm]	5.6 ± 2.7	3.4 ± 1.8	4.2 ± 2.2	< 0.001	ANOVA
Stage				< 0.001	chi-square
pT1a	941 (31.8%)	859 (77.5%)	135 (51.3%)		
pT1b	1033 (35.0%)	199 (18.0%)	92 (35.0%)		
pT2	489 (16.5%)	26 (2.3%)	15 (5.7%)		
pT3a	492 (16.6%)	24 (2.2%)	21 (8.0%)		
Grade				< 0.001	chi-square
G1/2	2468 (87.6%)	1024 (93.6%)	233 (93.6%)		
G3/4	350 (12.4%)	70 (6.4%)	16 (6.4%)		
Histological subtype				< 0.001	chi-square
ccRCC	2492 (85.8%)	846 (76.6%)	207 (79.6%)		
Non-ccRCC	411 (14.2%)	258 (23.4%)	53 (20.4%)		

^1^At the time of renal surgery. Abbreviations: SD = standard deviation, RN = radical nephrectomy, ePN = elective partial nephrectomy, iPN = imperative partial nephrectomy, RCC = renal cell carcinoma, RCC, ccRCC = clear cell RCC.

### Correlation of the surgical approach with patient/tumor characteristics

ePN was performed in 1108 (25.6%), iPN in 263 (6.1%) and RN in 2955 (68.3%) patients. Tumors were significantly better differentiated and smaller in the PN than in the RN subgroup (Table 1). Accordingly, patients with early stage RCC were treated by PN significantly more often than those with higher tumor stages (p < 0.001, chi^2^ test, Table 1). Non-clear cell RCC was found more frequently in patients submitted to ePN and iPN than in those undergoing RN (Table 2). Interestingly, non-clear cell tumors were not significantly smaller than clear cell RCC (mean, 4.88 vs. 4.95 cm, p = 0.57, t-test). Among all evaluable patients, 7.6% in the ePN, 16.1% in the iPN, and 27.2% in the RN subgroup presented with clinical symptoms at the time of diagnosis (p < 0.001, chi^2^ test).

Finally, patients who underwent ePN were significantly younger than those submitted to RN (Table 1). Surprisingly, more men than women (28.0% vs. 21.8%; p < 0.001, chi^2^ test) were treated by ePN, even though the mean tumor diameter did not differ significantly between men and women (4.9 vs. 5.0 cm; p = 0.17, t-test) Table 2.

**Table 2:** Factors that influenced the type of surgery (ePN vs. RN) in patients with pT ≤ 3a RCC and no detectable metastasis at the time of surgery using multivariate regression analysis

**Variable**	**P value**	**OR (95% CI)**
Age [in years]^1^	< 0.001	0.98 (0.97-0.98)
Sex	0.14	
Female		1 (Reference)
Male		1.15 (1.96-1.39)
Histological subtype	< 0.001	
ccRCC		1 (Reference)
Non-ccRCC		1.85 (1.47-2.33)
Differentiation	< 0.001	
G1-2		1 (Reference)
G3-4		0.53 (0.38-0.73)
Tumor diameter [in cm]	< 0.001	0.52 (0.49-0.56)
Medical center	< 0.001	
Time of surgery [in years]	< 0.001	1.06 (1.04-1.08)

RCC = renal cell carcinoma, ccRCC = clear cell RCC, G = tumor grade, RN = radical nephrectomy, ePN = elective partial nephrectomy, RN = radical nephrectomy.

### Association between tumor/patient characteristics, the type of surgery, and overall survival

**Figure 1:**
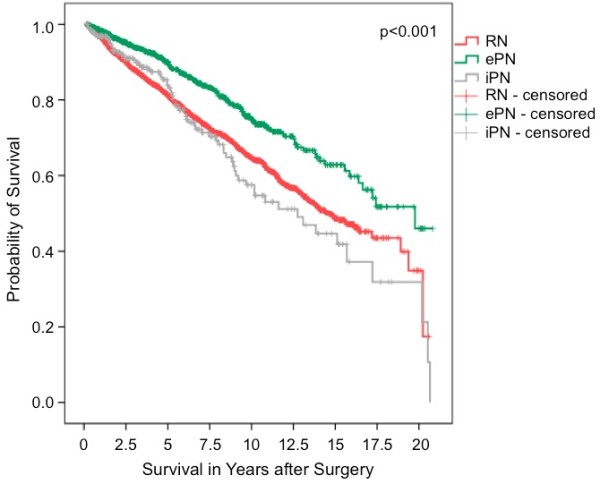
**Overall survival (Kaplan-Meier) for patients with pT ≤ 3a RCC and no detectable metastasis at the time of surgery plotted against the surgical procedure.** The 5- and 10-year survival rates of all evaluable patients were 81.2% and 64.7% for RN (n = 2936), 90.0% and 74.6% for ePN (n = 1103), and 83.9% and 57.5% for iPN (n = 261) (p < 0.001, log rank).

Using univariate Cox regression analysis we could reveal that in contrast to male sex (HR 1.04, 95% CI 0.92-1.18, p = 0.51) and non-clear cell histology (HR 0.93, 95% CI 0.79-1.10, p = 0.40), the following were significantly associated with reduced OS: age (in years, HR 1.05, 95% CI 1.04-1.06, p < 0.001), tumor stage (p < 0.001), increasing tumor diameter (in cm, HR 1.08, 95% CI 1.06-1.10, p < 0.001), and poor tumor differentiation (HR 2.10, 95% CI 1.76-2.50, p < 0.001). The type of tumor surgery also correlated significantly with OS: unlike iPN (HR 1.12, 95% CI 0.88-1.43, p = 0.35), ePN was associated with a significantly longer OS than RN (HR 0.61, 95% CI 0.51-0.71, p < 0.001). The Kaplan-Meier 5- and 10-year OS rates were 90.0% and 74.6% for ePN, and 81.2% and 64.7% for RN (p < 0.001; log rank; Figure 1).

To further assess the potential advantage of ePN over RN, we separately evaluated patients with tumors ≤ 4 and > 4 cm. In either case ePN was associated with significantly improved OS (HR 0.68, 95% CI 0.55-0.85, p = 0.001 for RCC ≤ 4 cm, and HR 0.69, 95% CI 0.51-0.93, p = 0.02 for RCC > 4 cm; univariate Cox regression).

The impact of age was also taken into consideration. A survival advantage of ePN over RN was found in all four calculated age subgroups (< 55, 55–63, 63–69, and > 69 years): HR 0.52 (95% CI 0.35-0.78), HR 0.70 (95% CI 0.51-0.96), HR 0.58 (95% CI 0.40-0.83), and HR 0.71 (95% CI 0.54-0.94; univariate Cox regression).

Finally, OS was superior with ePN vs. RN in all 5 periods evaluated: < 1990: HR 0.80 (95% CI 0.57-1.12), 1990–1994: HR 0.62 (95% CI 0.46-0.84), 1995–1999: HR 0.51 (95% CI 0.36-0.72), 2000–2004: HR 0.57 (95% CI 0.39-0.84), ≥ 2005: HR 0.56 (95% CI 0.27-1.13).

### Parameters affecting overall survival in multivariate analysis

Multivariate analysis confirmed that in our population of patients with pT ≤ 3a RCC and no detectable metastasis at the time of renal surgery, the type of surgery was an independent prognosticator for OS; ePN versus RN was associated with a hazard ratio of 0.79 (95% CI 0.66-0.94, p = 0.008, Cox regression; Table 3). We subsequently repeated our multivariate analysis with stepwise exclusion of different medical centers to avoid a bias caused by divergent results from individual hospitals. The hazard ratio for ePN varied but was significantly < 1.0 (range: 0.61-0.89) for all possible calculations.

**Table 3:** Multivariate analysis identified elective nephron-sparing surgery (ePN) as an independent prognostic factor for overall survival in patients with pT ≤ 3a RCC and no detectable metastasis at the time of surgery including all participating centers

**Variable**	**P value**	**HR (95% CI)**
Age [in years] ^1^	< 0.001	1.06 (1.05-1.06)
Sex	0.005	
Female		1 (Reference)
Male		1.21 (1.06-1.39)
Histological subtype	0.25	
ccRCC		1 (Reference)
Non-ccRCC		0.90 (0.76-1.07)
Differentiation	< 0.001	
G1-2		1 (Reference)
G3-4		1.88 (1.57-2.26)
Tumor diameter [in cm]	< 0.001	1.07 (1.04-1.09)
Surgical procedure	0.02	
RN		1 (Reference)
ePN	0.008	0.79 (0.66-0.94)
iPN	0.62	1.07 (0.83-1.38)
Year of surgery [in years]	0.01	0.99 (0.98-0.99)

^1^At the time of surgery. RCC = renal cell carcinoma, ccRCC = clear cell RCC, G = tumor grade, RN = radical nephrectomy, ePN = elective partial nephrectomy, iPN = imperative partial nephrectomy.

## Discussion

For many decades, RN was regarded as standard therapy for localized RCC [25]. This dogma has changed dramatically in recent years [1] because a large number of studies have shown that PN for elective or imperative indications is a technically feasible and safe surgical procedure with an only slightly higher complication rate [6,8,26]. Moreover, the oncological outcomes appear to be fully comparable in terms of local relapses and tumor-specific survival [4,6,24]. The main advantage of PN, however, lies in the markedly lower rate of postoperative renal failure achieved by preserving functional nephrons [18,19,21,24]. Thus, nephron sparing surgery-especially ePN-is now considered the gold standard for treatment of small renal tumors [1,9].

Several non-randomized studies have recently described a longer OS for PN than for RN in patient populations with mostly small renal tumors [14,15,27,28]. This was attributed to lower rates of surgery-associated chronic renal failure and subsequent CVD. However, these results do not go unchallenged [21], and the studies largely show the same limitations: retrospective and not comprehensive data collection, mostly small patient populations, and possible selection bias. Moreover, it has not yet been fully clarified whether surgically induced renal failure, like the type of chronic renal failure due to various internal disorders, also involves an increased risk of CVD and subsequent mortality [22,29].

Up to now, there has been only one randomized phase III study (EORTC-GU 30904) comparing the safety and effectiveness of ePN and RN in patients with localized renal tumors [24]. The 541 patients included had renal tumors up to 5 cm in diameter. During a median follow-up of more than 9 years, no survival advantage was found for patients who underwent PN. On the contrary, the intent-to-treat population had a calculated 10-year OS rate of 81.1% for RN and only 75.7% for ePN, and the PN group did not have fewer cardiovascular events (9.3% vs. 7.3%). Tumor-specific survival was nearly equal. Limitations of the study include the involvement of 45 varyingly experienced centers in 17 countries and the failure to recruit the intended sample size (1300 patients). Nevertheless, this randomized investigation was unable to demonstrate a survival advantage for PN. Since these results were generally surprising and did not appear to be scientifically conclusive, we found it expedient to evaluate another very large patient population that was treated at 6 German university medical centers. Unlike the EORTC trial [24], our retrospective study only included patients with histologically verified RCC. Since those who underwent ePN in our patient population had a younger median age as well as smaller and better differentiated tumors, it was not surprising that they had a significantly longer median OS than those submitted to RN. However, subsequent multivariate analysis also showed a significant survival advantage for the ePN group.

Even though we are dealing with a retrospective analysis that does not include comorbidity, this large study shows that OS appears to be better, but by no means worse, for ePN than for RN. Of great interest is also the finding that patients who underwent nephron-sparing surgery for imperative indications did not have a significantly worse prognosis than the RN group, even though they probably tended to have more serious comorbidities.

Therefore, our results are in line with a recent meta-analysis published by Kim et al. [30] who also found that PN lead to a 19% risk reduction in all-cause mortality. However, in contrast to our study evaluating a rather homogeneous patient collective with surgically resected RCC, Kim et al. [30] included patients with benign tumors and patients from observational studies.

Our results also support current recommendations to perform a PN for small renal tumors. However, in line with other recent publications [5,10-13,31], we were able to demonstrate in subgroup analyses that PN may also be justified for larger tumors leading to a potentially better prognosis.

Hillyer et al. [32] recently described that even elderly RCC patients do not have a significantly increased perioperative risk if submitted to organ-sparing surgery and benefit accordingly from PN. Smaldone et al. [33] demonstrated a survival advantage of PN over RN for patients up to age 85. Chang et al. [34] examined the secondary economic impact of RN compared to PN on the U.S. health care system. They came to the conclusion that PN is associated with fewer follow-up costs than RN irrespective of age, which also reflects the lower postoperative morbidity rate in patients treated by PN. We divided our patient population into 4 subgroups to assess the influence of age on the prognostic relevance of the surgical procedure. No significant difference was found between the subgroups: ePN was associated with a lower risk of shorter OS irrespective of age.

However, our study also has significant limitations. It is a retrospective non-randomized study in which patients’ comorbidities and general condition could not be taken into account. There was also a lack of detailed information on preoperative renal function. Moreover, there was no stringent standardization of criteria for the selection of the surgical approach and no central pathological review. Thus, a significant selection bias cannot be excluded. Only recently, Shuch et al. [35] published a highly interesting matched cohort study using the Surveillance, Epidemiology, and End Results (SEER)-Medicare data base. Elderly patients treated with PN or RN for localized RCC (≤ 4 cm) were compared with two control groups (non–muscle-invasive bladder cancer and noncancer controls). Median overall survival following PN was significantly higher than in controls without cancer or with nonmuscle invasive bladder cancer, while median overall survival after RN was similar among the 3 groups, confirming the hypothesis that selection bias may be present in observational data and that RN may be less harmful than often believed [35,36].

On the other hand, our study stands out in that we could assess whether PN was performed for elective or imperative indications even though this definition was not based on hard criteria but the individual subjective surgeon’s appraisal (based on the number of kidneys, renal function and comorbidities). Remarkably, the iPN group showed a poorer prognosis than the ePN group, as expected, but not a significantly worse OS than the RN group. At the same time, it can at least be speculated that the patients submitted to iPN probably tended to have the highest comorbidity in our patient population. A last limitation is that a tumor-specific survival analysis could not be performed for lack of reliable cause-of-death information.

Taken together, our results suggest that PN is associated with a potentially better but apparently not worse OS irrespective of center, age, and tumor size. This could of course only be definitively confirmed by an adequately large randomized study that provides detailed assessment of the patients’ general condition, comorbidities including renal function, and tumor-specific characteristics; a valid nephrometry score might also be taken into account [37-39]. However, it seems doubtful at present whether such a study a) would achieve better patient recruitment than the already prematurely terminated EORTC 30904 trial and b) would still be ethically acceptable, at least for patients with small tumors. There is also the question of what could possibly argue against performing a PN, whether for elective or imperative indications, considering that the rate and severity of postinterventional chronic renal failure could be significantly reduced, while perioperative morbidity is not significantly increased, and oncological outcomes are comparable to those of RN. Is this alone not reason enough to favor PN, regardless of whether or not the surgically induced chronic renal failure is directly associated with the occurrence of CVD? In any case, the results of our large multicenter study support the performance of PN in all cases where it appears technically feasible and oncologically safe.

## Conclusion

Even allowing for the significant weaknesses and selection bias any retrospective analysis may involve, our results indicate that in patients with localized RCC, PN may be associated with better OS than RN. Patients with RCC > 4 cm as well as elderly patients did also seem to benefit from a nephron-sparing approach.

## Competing interests

The authors declare that they have no competing interests.

## Authors’ contributions

FCR, SaSt, KJ, MJ and AJS carried out the data acquisition, participated in the data interpretation and drafting of the manuscript. AJS performed the statistical analysis. FB, GW, JS, TJS and HW carried out the data acquisition. WB, MS, RH, JWT, StSi, AH, MS and MAK revised the manuscript for important intellectual content. AJS, FCR, MJ and SaSt designed and supervised the study. All authors read and approved the final manuscript.

## Pre-publication history

The pre-publication history for this paper can be accessed here:

http://www.biomedcentral.com/1471-2407/14/372/prepub
